# Which risk factors significantly influence the outcome of traumatic brain injured patients with alcohol use disorder?

**DOI:** 10.1007/s00068-023-02346-1

**Published:** 2023-08-14

**Authors:** Dorothee Cäcilia Spille, David Kuroczik, Dennis Görlich, Julian Varghese, Michael Schwake, Walter Stummer, Markus Holling

**Affiliations:** 1 Department of Neurosurgery, University Hospital Münster, Münster, Germany; 2 Institute of Biostatistics and Clinical Research, University of Münster, Münster, Germany; 3 Institute of Medical Informatics, University of Münster, Münster, Germany

**Keywords:** GOS, Alcohol, Traumatic brain injury, Risk factors

## Abstract

**Purpose:**

Every year, approximately 10 million people worldwide suffer a traumatic brain injury that leads to hospitalization or mortality. Chronic and acute alcohol intoxication increase the risk of developing traumatic brain injury. Alcohol use disorder (AUD) as a predictor of outcome in neurosurgical patients and the definition of risk factors have been sparsely addressed so far. This study aims to improve the understanding of the effects of alcohol use disorder in the context of neurosurgical therapy.

**Methods:**

This study included patients admitted to Münster University Hospital with a traumatic brain injury and alcohol use disorder from January 1, 2010, to December 31, 2018. Univariate and multivariate analyses were performed to identify risk factors for a poorer outcome, assessed by the Glasgow Outcome Score.

**Results:**

Of the 197 patients included, 156 (79%) were male, and 41 (21%) were female, with a median age of 49 years (IQR 38–58 years). In multivariate analyses, age (*p* < 0.001), the occurrence of a new neurologic deficit (*p* < 0.001), the development of hydrocephalus (*p* = 0.005), and CT-graphic midline shift due to intracerebral hemorrhage (*p* = 0.008) emerged as significant predictors of a worse outcome (GOS 1–3). In addition, the level of blood alcohol concentration correlated significantly with the occurrence of seizures (*p* = 0.009).

**Conclusions:**

Early identification of risk factors in patients with alcohol use disorder and traumatic brain injury is crucial to improve the outcome. In this regard, the occurrence of hydrocephalus or seizures during the inpatient stay should be considered as cause of neurological deterioration in this patient group.

## Introduction

Every year, approximately 10 million people worldwide suffer from traumatic brain injury that leads to hospitalization or mortality [1]. Likewise, alcohol use disorder (AUD), defined by DSM-5 criteria, is one of the most prevalent mental disorders worldwide [2]. Both chronic alcohol abuse and acute alcohol intoxication increase the risk of developing traumatic brain injury [3, 4] and aggravates the severity of the trauma [5]. Approximately 30%-64% of persons are involved in trauma yearly due to alcohol use disorder [6, 7]. Due to a high number of unreported cases of alcohol use, a greater percentage of those affected can be assumed. There has also been an increase in alcohol use disorder in crises, such as the recent COVID pandemic, so this topic can be regarded as up-to-date and relevant [8–10].

In this context, three critical factors influence the assessment of the severity of traumatic brain injury. Firstly, the patient’s lack of ability to respond, resulting in a lack of protective reflexes to the trauma mechanism [11], as well as the impairment of the amygdala reactivity to social signals of threat [12]. Secondly, the patients have limited mnestic capacity as well as reduced compliance regarding the neurological assessment. This often results in an initial incorrect estimation of the clinical condition, e.g., according to GCS [4]. Thirdly, patients with chronic alcohol consumption have a higher risk of liver cirrhosis/liver damage, which may lead to impaired synthesis of coagulation factors and may result in an increase in overall morbidity [13, 14].

This study aimed to specify pre- and postoperative clinical and radiological risk factors in patients with traumatic brain injury and alcohol use disorder to address the impact on neurosurgical treatment decisions and optimize rehabilitation. Although alcohol use disorder can be widely detected in patients with traumatic brain injury, few studies have addressed risk factors for poorer outcomes in this patient population. Nonetheless, the need for a more comprehensive understanding of this patient group is apparent.

## Materials and methods

### Study design

In this retrospective study, 197 patients, who were transferred with a traumatic brain injury to Münster University Hospital, a level 1 trauma center, between January 1, 2010, and December 31, 2018, were included. Patients were screened by searching the hospital information system by the following ICD-10 codes: toxic effect of ethanol, intracerebral hemorrhage, subdural hematoma, epidural hematoma, traumatic subarachnoid hemorrhage, and traumatic brain injury. The traumatic brain injuries were classified as severe, moderate, and mild. Intracerebral trauma injury on CT imaging was not obligatory for inclusion in this study. In addition, patients with an elevated blood alcohol concentration in the laboratory chemical control and/or patients with moderate to severe AUD as defined by the DSM-5 (American Psychiatric Association, DSM-5 Task Force, 2013), were included. A clear distinction between acute and chronic alcohol abuse of the patients was not possible and was not performed on our part following the DSM-5 classification, with which, in contrast to the previous division into abusive/harmful use and the dependence diagnosis, the single disorder diagnosis “alcohol use disorder” was established. Patients without a distinct alcohol history were excluded. All patients were admitted through the hospital's emergency trauma room and received a CT trauma scan. The local ethics committee approved the study (2019-387-f-S).

### Variables

Demographic, clinical, and radiological data were collected for each patient and included clinical information at the time of admission (age, sex, anisocoria, disorientation, unconsciousness, retrograde amnesia), laboratory chemistry values at the time of admission (thromboplastin time (PTT), prothrombin time (PT), blood alcohol level), trauma mechanism (traffic accident, exposure to violence, alcohol abuse, unclear trauma mechanism), CT-graphically manifest diagnosis (skull fractures, epidural hematoma, subdural hematoma, traumatic subarachnoid hemorrhage, intracerebral hemorrhage), duration of treatment (ventilator hours, length of hospital stay), postoperative complications as secondary outcomes (meningitis, abscess, hydrocephalus, delirium, seizures, urinary tract infection, postoperative secondary bleeding) and comorbidities at admission (cardiovascular diseases, diabetes, hyper-/hypothyroidism, liver diseases, depression, psychosis). In addition, based on both the initial CT and the first CT follow-up at approximately six hours, midline shift was measured using PACS DICOM (Digital Imaging and Communications in Medicine) viewer.

### Outcome

The Glasgow Coma Scale, as an internationally established score for acute assessment of the severity of brain dysfunction, was applied for the initial assessment of the severity of traumatic brain injury, according to our hospital's standard.

The Glasgow Outcome Scale, as primary outcome, was used to assess prognosis and recovery after traumatic brain injury. [15, 16] It subdivides the following five categories: (1) death, (2) persistent vegetative state, (3) severe disability, need for support 24 h a day, (4) moderate disability, independent but disabled, (5) good recovery. We dichotomized the scale into poor (GOS 1–3) and regular convalescence (GOS 4–5). The Glasgow Outcome Scale was determined at discharge and after a 6-month time interval.

### Statistics

Data were described by standard statistics, e.g., median and interquartile range and mean and standard deviation as well as absolute and relative frequencies for continuous and categorical variables, and compared by Mann–Whitney *U* and Fisher’s exact test, respectively. The univariate logistic regression model was used to determine risk factors for a worse outcome. The dependent variable in this analysis was GOS. The variables with significant results in the univariate regression model were included in the multivariate regression model. Odds ratios (ORs) with 95% confidence intervals (CIs) were calculated. All variables from the final forward model were selected for the backward model. All reported p values are two-sided. A *p* value of < 0.05 was considered to be statistically significant. Statistical analysis was performed using SPSS (IBM Corp. Released 2016. IBM SPSS Statistics for Windows, Version 24.0. Armonk, New York, USA).

## Results

### Clinical and radiological characteristics

Of the 197 patients included, 156 (79%) were men, and 41 (21%) were women with a median age of 49 years (IQR 38–58 years). Blood alcohol level was determined on admission (in patients suspected of consuming alcohol). Of these, 13 patients had a negative control. Patients who tested positive for alcohol showed a mean blood alcohol concentration of 1.80 per mille (SD 1.05 per mille). In the first laboratory control, a median PT value of 99% (IQR 90–108%; ref.: 70–130%) and a median PTT value of 29 s (IQR 27–32 s.; ref.: 29–38 s) could be determined. Of the 184 patients with available laboratory sampling, 8 patients (4%) showed decreased PT values, and 11 patients (5%) showed increased PTT values. The patient population had cardiovascular comorbidities in 29% of the cases (*n* = 58), pulmonary pathologies in 31% (*n* = 61), hepatic pathologies in 15% (*n* = 29), known diabetes mellitus in 3% (*n* = 6) and pathologies of the thyroid gland in 8% of the patients (*n* = 16), respectively. In addition, 14 patients (7%) showed depression, and 27 patients (15%) developed psychosis.

In most patients, the trauma mechanism was unclear due to unconsciousness and lack of witnesses (104 patients; 53%). Traffic accidents could be identified as the cause of trauma in 21% of the cases (*n* = 41). The trauma mechanism in 32 patients (16%) could be attributed to alcohol use disorder. Twelve patients (6%) were hospitalized due to an assault. 50% of the patients arrived at the emergency room as secondary transfers from an external hospital.

On admission, a GCS of 13–15 could be documented in 62% of patients, a GCS of 9–12 in 14%, and a GCS of 3–8 in 24%, respectively. In addition, 18 of the patients (9%) showed anisocoria. Disorientation was present in 30% of the patient group, 26% were unconscious, and 20% presented amnesia.

Subsequent CT trauma scans revealed skull fractures in 43% and facial fractures in 19% of the cases. The intracranial hemorrhage distribution was as follows: 11 patients with epidural hematomas, 37 patients with subdural hematomas, 33 cases with the formation of traumatic subarachnoid hemorrhage, and 23 cases with intracerebral hematomas. In median, no midline shift could be measured in the first CT scan (range 0 to 20 mm). The same applies to the measurements after 6 h in the CT follow-up. Cerebrovascular pathologies were detected as incidental findings in 4.6% of patients based on angiography.

### Therapy and adverse events

Approximately half of the patients (48.7%; *n* = 106) with traumatic brain injury received neurosurgical treatment. In the majority of cases, an external ventricular drain was placed (40.6%, *n* = 80). 36 patients (18.3%) received hematoma evacuation via a small craniotomy, while 23 (11.7%) underwent decompressive hemicraniectomy. Revision surgery was required in 41 patients (21%) due to intracranial rebleeding.

The mean hospital length of stay was 9 days (SD 10 days), with a mean stay of one day (SD 1 day) in the intensive care unit. The duration of ventilation for the patients ranged between 0 and 850 h (mean 26; SD 92 h). Postoperative complications included the occurrence of a new neurologic deficit in 47 patients. In addition, 4% of the cases had urinary tract infections, 12 patients intracranial infections in the form of meningitis in 5 patients and formation of brain abscess in 3 patients. 5% of the patient group developed postoperative hydrocephalus. The occurrence of seizures was documented in 38 patients, and the development of delirium in 35 patients. Baseline data, including comorbidities, are depicted in Table 1.

**Table 1:** Baseline data, including comorbidities

Variable	*n*	%
Sex		
Male	156	79
Female	41	21
Age	Median: 49 (IQR 38–58)	
First blood alcohol level (‰)	Mean: 1.80 (SD 1.05)	
First PT (%)(reference: 70–130)	Median: 99 ( IQR 90–108)	
First PTT (sec)(reference: 29–38)	Median: 29 ( IQR 27–32)	
Comorbidities		
Psychosis	27	14
Depression	14	7
Cardiovascular diseases	58	29
Pulmonary diseases	61	31
Hepatic pathologies	29	15
Diabetes	6	3
Thyroid gland pathology	16	8
Trauma mechanism		
Unknown injury mechanism	104	53
Traffic accident	41	21
Violent attacks	12	6
C2-related trauma	32	16
Clinical presentation		
Anisocoria	18	9
Disorientation	59	30
Unconsciousness	51	26
Amnesia	39	20
TBI grade		
Mild	123	62
Medium	27	14
Severe	47	24
Diagnosis on initial CT		
Facial fracture	38	19
Skull fracture	84	43
Epidural hematoma	11	6
Subdural hematoma	37	19
Traumatic SAH	33	17
Intracerebral hemorrhage	23	12
Non skull fractures	26	13.2
Midline shift at hospital admission (mm)	44	22.3
	Mean 1.9 ( SD 4.4)	
Midline shift at first control (mm)	25	12.7
	Mean: 0.8 ( SD 2.8)	
Cerebrovascular disease	9	4.6
Neurosurgical operations	106	48.7
External ventricular drainage	80	40.6
Craniotomy	36	18.3
Decompressive craniectomy	23	11.7
Trauma or general surgery	17	8.6
Adverse events		
New neurological deficit	47	24
Meningitis	9	5
Abscess	3	2
Hydrocephalus	10	5
Delirium	35	18
Seizures	38	19
Urinary tract infection	8	4
Postoperative bleeding	41	21
Interhospital transfer	98	50
Stay (days)	Mean: 9 ( SD 10)	
Stay in ICU (days)	Mean: 0.9 ( SD 1.0)	
Mechanical ventilation (hours)	Mean 26.2 ( SD 92.3)	
GOS at discharge		
1	4	2
2	4	2
3	43	22
4	46	23
5	77	39
GOS at discharge		
3-Jan	51	29
5-Apr	123	71
GOS after 6 months		
3-Jan	6	3
5-Apr	58	30
Death	4	2

### Follow-up

The Glasgow Outcome Score at discharge was 4–5 in 123 patients (71%) and 1–3 in 51 patients (29%). 6% of patients left the clinic against medical advice. Of the 64 patients who presented again after a time interval of 6 months, 3% (*n* = 6) had a poor outcome (GOS 1–3). Four patients died. Incidental brain metastases were detected in CT diagnostics in four patients (2%).

### Univariate analyses for the prediction of risk factors for poorer outcome (GOS 1–3)

The results of the univariate analyses are presented in Table 2. In summary, male patients showed a worse outcome at discharge than female patients (OR 2.22, 95% CI 1.04–4.73; *p* = 0.038). A lower GCS at admission (OR 2.18, 95% CI 1.12–4.24, *p* = 0.021) and higher age (OR 1.06, 95% CI 1.04–1.09, *p* < 0.001) were significantly associated with a lower GOS value, as well. Cardiovascular (OR 0.31, 95% CI 0.15–0.63, < 0.001) and pulmonary (OR 0.13, CI 0.06–0.26, *p* < 0.0001) pre-existing conditions revealed to be critical preoperative comorbidities for worse outcome. Amnesia for the course of the accident (OR 6.62, 95% CI 1.93–22.63, *p* = 0.003), but interestingly nor unconsciousness (*p* = 0.544) neither disorientation (*p* = 0.166) correlated significantly with a lower GOS. Presentation of an intracerebral hematoma on CT imaging (OR 0.16, 95% CI 0.06–0.42, *p* < 0.001) and the resulting midline shift (OR 1.12, 95% CI 1.05–1.20, *p* < 0.00) were both predictors of a worse outcome. Indication for a neurosurgical operation was related to a lower GOS (OR 3.84, 95% CI 2.27–6.50, *p* < 0.0001). A longer duration of surgery (OR 1.01, 95% CI 1.00–1.01, *p* < 0.0001), duration of ventilation (OR 1.02, 95% CI 1.01–1.03, *p* < 0.001), and generally longer duration of inpatient stay (OR 1.10, 95% CI 1.05–1.15, *p* < 0.0001) were significantly associated with a worse outcome at discharge, as well. Furthermore, adverse events such as a new neurological deficit (OR 0.13, 95% CI 0.06–0.30, *p* < 0.0001), the occurrence of meningitis (OR 0.05, 95% CI 0.01–0.43, *p* = 0.006), hydrocephalus (OR 0.04, 95% CI 0.01–0.36, *p* = 0.004), delirium (OR 0.25, 95% CI 0.12–0.56, *p* = 0.001, the development of an epileptic seizure (OR 0.33, 95%: 0.15–0.72, *p* = 0.006) or a postoperative rebleeding (OR 0.25, 95% CI 0.12–0.52, *p* < 0.001) could be identified as predictors of a worse outcome during the inpatient stay.

**Table 2:** Univariate logistic regression analysis for prediction of a low Glasgow Coma Outcome Score (GOS 1–3)

Variable	GOS 1–3 (0)	GOS 4–5 (1)	OR (95%CI), *p *value
Sex			
Male	35 (26%)	102 (74%)	2.22 (1.04–4.73); **0.038**
Female	16 (43%)	21 (57%)	
Age (years)	Median: 58	Median: 47	1.06 (1.04–1.09); < **0.001**
	IQR 47–73	IQR 35–55	
TBI grade			
Mild (GCS 13–15)	23 (45.1%)	79 (64.2%)	2.18 (1.12–4.24);** 0.021**
Medium (GCS 9–12)	8 (15.7%)	18 (14.6%)	0.80 (0.33–1.92); 0.618
Severe (GCS 3–8)	20 (39.2%)	26 (21.1%)	0.41 (0.20–0.84); **0.015**
First blood alcohol level (‰)	Median: 1.60	Median: 1.90	0.69 (0.46–1.03); 0.071
	IQR 0.35–2.25	IQR 1.50–2.60	
Comorbidities			
Psychosis	10 (19.6%)	15 (12.2%)	0.57 (0.24–1.37); 0.208
Depression	4 (7.8%)	8 (6.5%)	0.82 (0.24–2.85); 0.751
Cardiovascular diseases	23 (45.1%)	25 (20.3%)	0.31 (0.15–0.63); **0.001**
Pulmonary diseases	34 (66.7%)	25 (20.3%)	0.13 (0.06–0.26);** < 0.0001**
Hepatic pathologies	10 (19.6%)	15 (12.2%)	0.57 (0.24–1.37); 0.208
Diabetes	2 (3.9%)	1 (0.8%)	0.20 (0.02–2.27); 0.194
Thyroid disease	6 (11.8%)	8 (6.5%)	1.06 (1.04–1.09)** < 0.0001**
Trauma mechanism			
Unknown injury mechanism	32 (62.7%	66 (56.3%)	0.35 (1.34–0.27); 0.272
Traffic accident	8 (15.7%)	41 (23.6%)	1.97 (0.83–4.63); 0.119
Violent attacks	2 (3.9%)	12 (6.9%)	2.17 (0.45–10.26); 0.329
C2-related trauma	8 (15.7%)	11 (8.9%)	0.53 (0.19–1.40); 0.200
Initial clinical presentation			
Anisocoria	8 (15.7%)	10 (10.3%)	0.47 (0.17–1.28); 0.143
Disorientation	20 (39.2%)	35 (28.5%)	0.61 (0.31–1.22); 0.166
Unconsciousness	16 (31.4%)	33 (26.8%)	0.80 (0.39–1.63); 0.544
Amnesia	3 (5.9%)	36 (29.3%)	6.62 (1.93–22.63); **0.003**
Diagnosis on initial CT			
Facial fracture	7 (13.7%)	31 (25.2%)	2.11 (0.86–5.18); 0.100
Skull fracture	12 (23.5%)	24 (66.7%)	0.78 (0.35–1.72); 0.552
Epidural hematoma	5 (9.8%)	5 (4.9%)	0.47 (0.13–1.62); 0.233
Subdural hematoma	15 (29.4%)	22 (17.9%)	0.52 (0.24–1.11); 0.093
Traumatic SAH	9 (17.5%)	24 (19.5%)	1.13 (0.48–2.63); 0.775
ICB	15 (29.4%)	8 (6.5%)	0.16 (0.06–0.42); < **0.001**
Non skull fractures	11 (21.6%)	13 (10.6%)	0.43 (0.17–1.03); 0.060
Initial MLS due to ICB (mm)	Mean: 4.20	Mean: 1.40	1.12 (1.05–1.20); < **0.001**
	SD 6.22	SD 3.57	
6 h control MLS due to ICB (mm)	Mean: 1.88	Mean: 0.50	0.86 (0.77–0.97); **0.013**
	SD 4.60	SD 1.75	
Cerebrovascular diseases	5 (9.8%)	4 (3.3%)	0.31 (0.08–1.20); 0.090
Number of neurosurgical operations	Median: 1	Median: 0	3.84 (2.27–6.50)**; < 0.0001**
	IQR 1–2	IQR 0–1	
Trauma or general surgery procedures	Median: 0	Median: 0	1.51 (0.73–3.11); 0.269
	IQR 0–0	IQR 0–0	
Surgery time (min)	Mean: 141	Mean: 52	1.01 (1.00–1.01); < 0**.0001**
	SD 151	SD 83	
Length of stay (days)	Mean: 16	Mean: 6	1.10 (1.05–1.15); < **.0001**
	SD 14	SD 7	
Duration of mechanical ventilation (hours)	Mean: 68	Mean: 7	1.02 (1.01–1.03); **0.001**
	SD 121	IQR 29	
Adverse events			
New neurological deficit	24 (47.1%)	13 (10.6%)	0.13 (0.06–0.30);** < 0.0001**
Meningitis	7 (13.7%)	1 (0.8%)	0.05 (0.01–0.43); **0.006**
Abscess	1 (2.0%)	1 (0.8%)	0.41 (0.03–6.68); 0.531
Hydrocephalus	8 (15.7%)	1 (0.8%)	0.04 (0.01–0.36); **0.004**
Delirium	18 (35.3%)	15 (12.2%)	0.25 (0.12–0.56); **0.001**
Seizures	16 (31.4%)	16 (13.0%)	0.33 (0.15–0.72); **0.006**
Postoperative bleeding	21 (41.2%)	18 (14.6%)	0.25 (0.12–0.52); < **0.001**

A *p* value of < 0.05 was considered to be statistically significant (in bold)

*TBI* traumatic brain injury, *SAH* subarachnoid hemorrhage, *MLS* midline shift, *ICB* intracranial bleeding, *IQR* interquartile range, *SD* standard deviation

The level of blood alcohol concentration at admission correlated only with the onset of seizures (*p* = 0.009) in univariate analysis.

### Multivariate analyses for the prediction of risk factors for poorer outcome (GOS 1–3)

In multivariate analyses, age (OR 0.95, 95% CI 0.92–0.97, *p* < 0.001), the extent of midline shift on initial CT due to intracerebral hemorrhage (OR 0.90, 95%CI:0.83–0.97, *p* = 0.008), the occurrence of a new neurological deficit (OR 0.14, 95% CI 0.06–0.36, *p* < 0.001) and the development of hydrocephalus (OR 0.04, 95% CI 0.00–0.38, *p* = 0.005) were found to be significant predictors of a low GOS at discharge (Table 3)*.*

**Table 3:** Multivariate logistic regression analysis for prediction of a worse outcome (GOS 1–3)

Variable	OR	95% CI	*p* value
Age	0.95	0.92–0.97	< 0.001
New neurological deficit	0.14	0.06–0.36	< 0.001
Hydrocephalus	0.04	0.00–0.38	0.005
Initial MLS due to ICB (mm)	0.90	0.83–0.97	0.008

Only statistically significant results (*p* < 0.05) are depicted

**Fig. 1:**
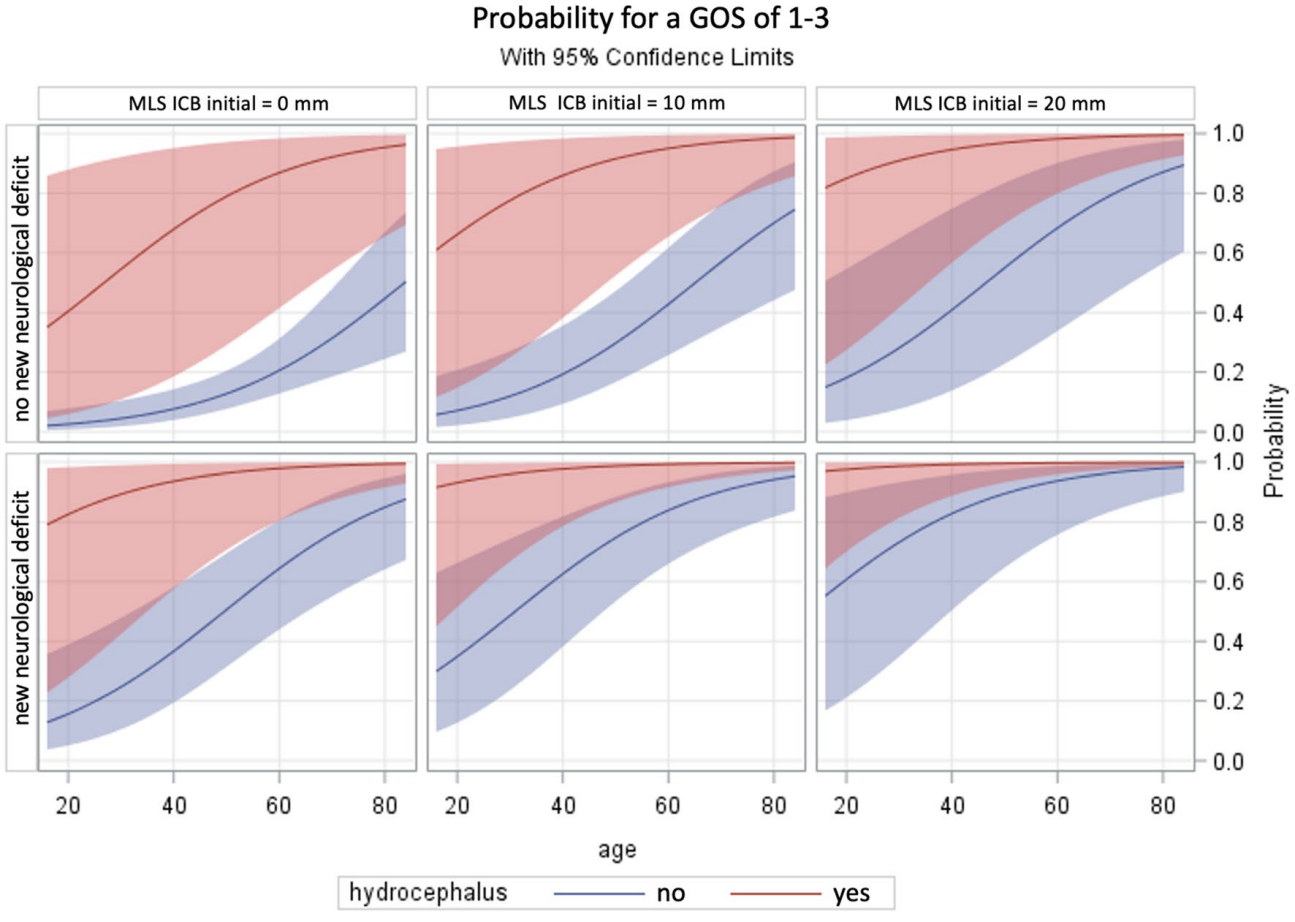
Schemes showing the probability of poor outcome (corresponding to GOS 1–3) based on the parameters significant in the multivariate analyses (hydrocephalus, new neurological deficit, midline shift due to intracerebral hemorrhage and age). For this purpose, the groups with and without the development of hydrocephalus and new neurologic deficit are presented separately. For each of these groups, the midline shift is shown as a function of age

The following Figs. 1 + 2 serve as an adjunct to a fast assessment of the patient's outcome in clinical practice.

*Remarkably, if both a new neurological deficit and hydrocephalus are present, the GOS amounts 1–3 regardless of age and CT findings. In the presence of only one risk factor, patients under 60 years of age have a superior outcome (*Fig. 1*).*

**Fig. 2:**
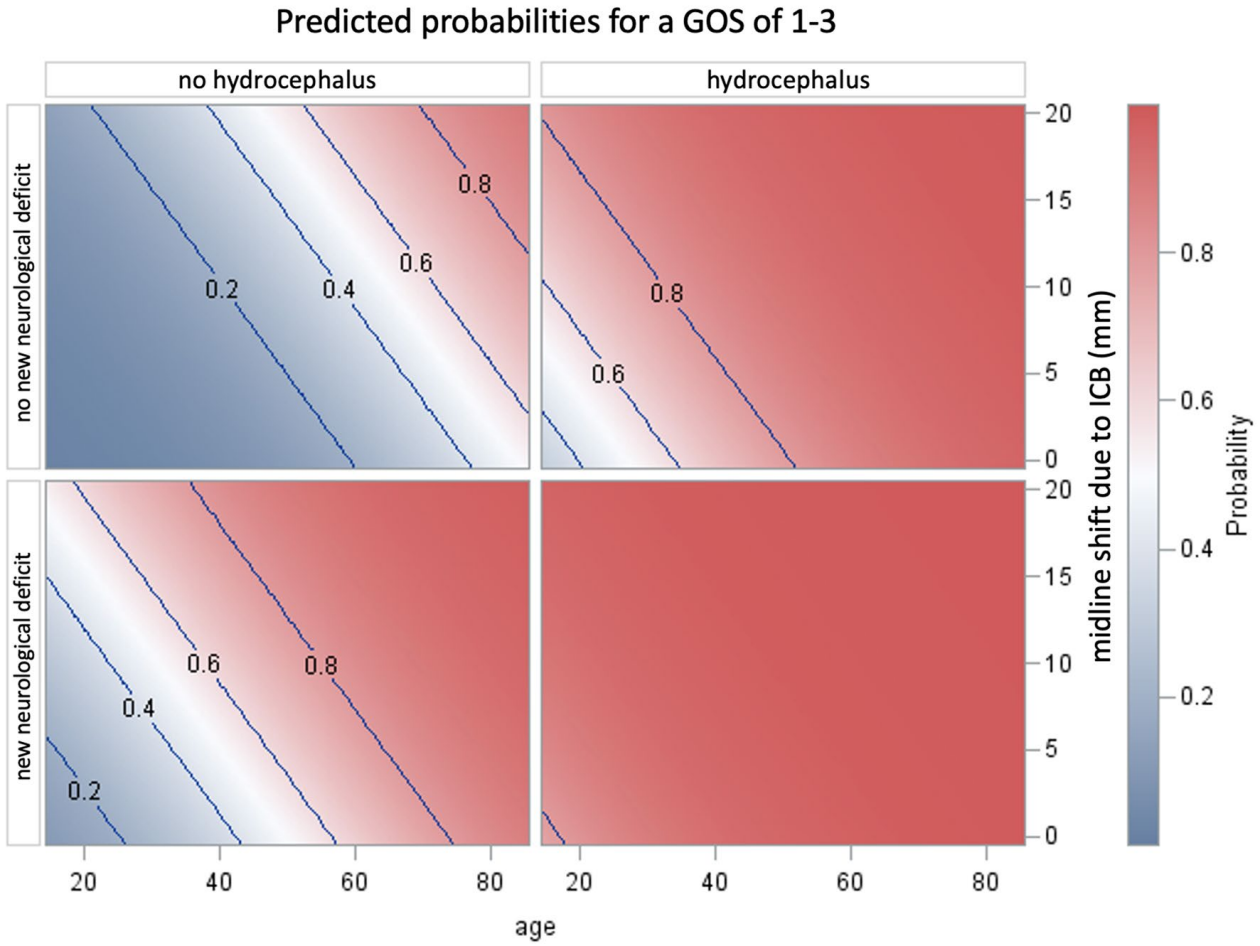
The patient cohort was classified, first, according to the development of a new neurological deficit (0 = absent; 1 = present) and, second, according to the extent of midline shift due to intracerebral hemorrhage (0 mm, 10 mm, and 20 mm). The graphs show the probability of a low Glasgow Outcome Score as a function of age. Separate curves were created for each of the patients with and without hydrocephalus

Furthermore, the extent of midline shift shows a significant influence on the outcome (Fig. 2). A 40-year-old patient with a marked midline shift of 2 cm on CT imaging has a probability of about 40% for a GOS of 1–3 if no severe adverse events occur. If he shows the development of a new neurological deficit during the inpatient course, the probability is 80% and increases to about 95%, if hydrocephalus develops (Fig. 2). In an 80-year-old patient with an extensive midline shift of about 2 cm, the probability of a poor outcome (GOS 1–3) is already 90%, with or without the development of a new neurological deficit or hydrocephalus.

## Discussion

The aim of this study was to define pre- and postoperative risk factors in patients with alcohol use disorder and identify corresponding patients at risk early in order to initiate preventive therapy. To date, few studies address improving the rehabilitative potential of this patient population, despite the fact that alcohol abuse is widespread and increases the risk of suffering a traumatic brain injury.

The majority of patients with an alcohol use disorder in our study were male (79%). This is consistent with the data in the literature [7, 13, 17]. Similarly, male patients had a worse outcome at discharge compared to female patients. This is certainly due to the generally lower life expectancy of the male population, who have a higher multimorbidity [18].

With regard to age, a wide confidence interval, ranging from 16 to 84 years, was striking in our patient cohort. As expected, the older patients showed a lower GOS at discharge than the younger ones. [17]

Interestingly, in our analysis, no influence of the blood alcohol level on the outcome could be detected. This is widely discussed in the literature [19]. On the one hand, an increased blood alcohol level is supposed to be associated with a worse outcome [20–22], but other studies could not confirm this [17, 23]. In a retrospective study with over 6000 trauma patients, Brockamp et al. investigated the impact of a high blood alcohol level and could not find any significant differences in prognosis between the patient groups with and without ethanol intoxication [13]. Some studies have shown that alcohol use disorder is associated with a worse outcome, whereas moderate consumption may lead to preconditioning [24]. This implies neuroprotective mechanisms against ischemic conditions through the induction of mild oxidative stress by an increase in reactive oxygen species (ROS) derived from NADPH oxidase [25]. Lin et al. performed an ethanol preconditioning followed by intracerebral hemorrhage induction in rats and reported an increased chaperone protein expression leading to reduced oxidative stress and proinflammatory cytokines release [24].

Furthermore, our study laboratory tests showed that only eight patients (4.3%) had a PT decrease (< 70%), and 11 patients (6.0%) had a PTT prolongation (> 38 s). Some studies described the influence of chronic liver injury from alcohol use disorder and consequently decreased synthesis of coagulation factors [14, 26, 27]. The low number of patients with laboratory deviations of coagulation factors in our cohort is most likely explained by the inclusion of a high number of patients with acute alcohol intoxication. This does not necessarily imply alcohol abuse over many years and chronic organ damage, such as liver cirrhosis. Likewise, the number of patients with liver diseases in our cohort is elevated at 15% compared with the general population [28]. Nevertheless, we could not identify this as a risk factor for a poor outcome, which contradicts the presence of end-stage liver diseases.

As expected, a low GCS at admission correlated with a lower GOS at discharge in the univariate analysis (*p* = 0.015). Similarly, other authors identified the initial GCS as a predictor of outcome [17].

Besides, in our study, cardiovascular and pulmonary diseases, as well as thyroid pathologies, had an influence on the further course of the patient’s recovery. Previous mental illnesses, in terms of depression or psychosis, had no impact. Davies and colleagues, however, observed the increased incidence of mental illnesses in patients with alcohol use disorder by performing a cross-sectional study including more than 38,000 participants from 13 different countries [8]. The influence of comorbidities on the outcome of patients with alcohol use disorder was likewise demonstrated by other studies [7, 29, 30].

Remarkably, the presence of amnesia had a decisive impact on the GOS at discharge (*p* = 0.003). This influence is reported, among others, by the colleagues Kosch et al. [31]. Over a period of 10 years, they examined a patient collective of over 600 persons with traumatic brain injury and were able to show a significant impact of the duration of amnesia on the length of inpatient stay as well as on the extent of the need for care measured by the Functional Independence Measure (FIM)-score.

While *extracerebral* hemorrhages, such as epidural and subdural hematoma, did not have a significant effect on outcome, the presence of *intracerebral* hemorrhage and the resulting midline shift was relevant for the patient’s clinical outcome at discharge. In contrast, other studies reported that the presence of a subdural or epidural hematoma adversely affected patient rehabilitation. In the Rotterdam CT score of traumatic brain injury [32], for example, midline shift is used as a predictor of worse outcome, without differentiation of the causality of midline shift. Furthermore, the presence of an epidural hematoma, as well as intraventricular and subarachnoid blood components, are included in the score. Regarding these parameters, we could not determine any influence on the outcome at discharge. We did not separately examine if compressed or absent basal cisterns were depicted on CT. To date, however, the extent of midline shift on CT or MRI has rarely been studied as a predictor of outcome. Asim et al. demonstrated that both the Rotterdam as well as the Marshall score could be used for prediction of outcome even in patients with alcohol use disorder [33].

Not surprisingly, the observation that a longer duration of surgery and increase in hospitalization time provide evidence for a lower GOS at discharge. This is consistent with the data collection of other authors [34].

Significant predictors for a worse outcome in patients with alcohol use disorder were especially postoperative adverse events. In particular, the formation of hydrocephalus and the development of a new neurological deficit showed an impact in the multivariate analyses. So far, Omran and colleagues demonstrated the influence of chronic alcohol consumption on ependymal cilia function [35]. Further studies showed the correlation between chronic alcohol use disorder and the occurrence of hydrocephalus [36, 37].

Subgroup analysis of our patient group showed that an elevated blood alcohol level on admission significantly correlated solely with the occurrence of seizures. An association between alcohol use disorder and seizures has been broadly reported in the literature [38–40] and is noteworthy high (approximately 50%) [41]. This should be considered, especially in intubated patients, as a cause of prolonged coma. Thus, systematic blood alcohol level sampling should be considered, especially in patients with a low GCS on admission as well as during the inpatient course.

In general, preliminary work can show that patients with an alcohol use disorder have a more impaired rehabilitation [42] as well as an increased risk of suffering further traumatic brain injuries [43]. Against this background, we consider the workup of an alcohol use disorder to be very relevant for prognostic assessment and initiation of preventive measures.

### Limitations

First of all, the data collection is retrospective. Secondly, blood alcohol concentration was not systematically determined in all trauma patients, and, with regard to alcohol use disorder, there was no precise definition of the extent and duration. It is assumed that there is a large number of unreported cases of potential alcohol use disorder among traumatized patients, some of whom cannot be amnestied. Therefore, we consciously refrained from using a control cohort. Furthermore, patients with both acute alcohol intoxication and chronic alcohol abuse were included, contributing to heterogeneity in the patient population. Arterial hypertension, as a relevant risk factor for the progression of intracerebral hemorrhage, was not included in our analysis, because the values were partly very fluctuating and, therefore, not completely reliable.

## Conclusions

The measurement of blood alcohol levels in patients with traumatic brain injury is recommended to be performed at a very low threshold, and the self- or foreign history of these patients should include questions referring to alcohol use disorder. Most notably, the development of hydrocephalus, age, midline-shift on CT due to intracranial bleeding, and the development of a new neurological deficit should be considered as potential risk factors for a worse outcome and need for extended rehabilitation programs. With increased alcohol levels, the occurrence of seizures should be kept in mind.

## Data Availability

The data supporting this study’s findings are available from the corresponding author, MH, upon reasonable request.

## Abbreviations

AUD: Alcohol use disorder
CT: Computer tomography
GCS: Glasgow Coma Score
GOS: Glasgow Outcome Score
ICB: Intracranial bleeding
ICU: Intermediate care unit
OR: Odds ratio
CI: Confidence interval
MLS: Midline shift
TBI: Traumatic brain injury
PTT: Thromboplastin time
PT: Prothrombin time
IQR: Interquartile range
SD: Standard deviation
